# Improvement of Medium Chain Fatty Acid Content and Antimicrobial Activity of Coconut Oil via Solid-State Fermentation Using a Malaysian *Geotrichum candidum*

**DOI:** 10.1155/2013/954542

**Published:** 2013-07-18

**Authors:** Anahita Khoramnia, Afshin Ebrahimpour, Raheleh Ghanbari, Zahra Ajdari, Oi-Ming Lai

**Affiliations:** ^1^Faculty of Food Science and Technology, Universiti Putra Malaysia, 43400 Serdang, Selangor, Malaysia; ^2^Faculty of Science and Technology, Universiti Kebangsaan Malaysia, 43600 Bangi, Selangor, Malaysia; ^3^Iranian Fisheries Research Organization, West Fatemi, Tehran 1411816616, Iran; ^4^Faculty of Biotechnology and Biomolecular Sciences, Universiti Putra Malaysia, 43400 Serdang, Selangor, Malaysia; ^5^Institute of Bioscience, Universiti Putra Malaysia, 43400 Serdang, Selangor, Malaysia

## Abstract

Coconut oil is a rich source of beneficial medium chain fatty acids (MCFAs) particularly lauric acid. In this study, the oil was modified into a value-added product using direct modification of substrate through fermentation (DIMOSFER) method. A coconut-based and coconut-oil-added solid-state cultivation using a Malaysian lipolytic *Geotrichum candidum* was used to convert the coconut oil into MCFAs-rich oil. Chemical characteristics of the modified coconut oils (MCOs) considering total medium chain glyceride esters were compared to those of the normal coconut oil using ELSD-RP-HPLC. Optimum amount of coconut oil hydrolysis was achieved at 29% moisture content and 10.14% oil content after 9 days of incubation, where the quantitative amounts of the modified coconut oil and MCFA were 0.330 mL/g of solid media (76.5% bioconversion) and 0.175 mL/g of solid media (53% of the MCO), respectively. MCOs demonstrated improved antibacterial activity mostly due to the presence of free lauric acid. The highest MCFAs-rich coconut oil revealed as much as 90% and 80% antibacterial activities against *Staphylococcus aureus* and *Escherichia coli*, respectively. The results of the study showed that DIMOSFER by a local lipolytic *G. candidum* can be used to produce MCFAs as natural, effective, and safe antimicrobial agent. The produced MCOs and MCFAs could be further applied in food and pharmaceutical industries.

## 1. Introduction

Coconut oil, which is a very important source of medium chain fatty acids (MCFAs), exhibits good properties due to its different metabolism pathway [1]. Three valuable MCFAs exist in coconut fat, namely, caprylic (C8:0), capric (C10:0), and lauric (C12:0) acids, where lauric acid makes up about 50% of the total FAs content. The antimicrobial effects of MCFAs against bacteria, fungi, viruses, and protozoa have been investigated extensively [2–5]. MCFAs are even preferable to polyunsaturated fatty acids (PUFAs), because some bacteria such as Lactobacilli are stimulated by the presence of these fatty acids [6]. Among MCFAs, lauric acid and its derivatives have been demonstrated as the most effective antimicrobial agents for foods and cosmetics. In addition, they are effective in alteration of ammonia concentration, methane production, and milk fatty acids composition of ruminants [4, 7–9]. Furthermore, Hristov et al. (2009) [9] showed that administration of free lauric acid and coconut oil together exhibits stronger antimicrobial effects compared to a single application.

Production of fatty acids from fats and oils is important due to its wide application as raw materials in food, cosmetic, pharmaceutical, and oleochemical industries [10]. The current techniques for production of fatty acids are based on chemical, physical, and enzymatic methods [11, 12]. The use of commercial lipases would be preferable due to the mild processing conditions and less energy used [12] however, it is not a cost-effective way at large scale. On the other hand, as stated by Sado Kamdem et al. (2008) [2], among the compounds naturally presented in high-fat foods, free fatty acids (FFAs) produced by lipolysis during storage can be regarded as potential bactericides and/or bacteriostatics. The limiting aspect of food fatty acids is generally due to their amount, which is lower than the minimal inhibitory concentration (MIC) and inactivation of pathogens [13]. Hence, effective and natural FFAs production needs to be improved to enhance the antimicrobial activity.

Fermentation is an important process to increase the availability of important nutrients by enzymatic hydrolysis of raw substrates especially in solid-state system (SSF). Filamentous fungi are the most widely applied microorganisms in SSF [14]. *G. candidum*, which is Generally Recognized As Safe (GRAS) [15, 16], has been employed in cheese industry for many years [15].

Many processes have been developed that utilize raw materials for the production of chemicals and value-added fine products [17, 18]. The application of coconut as solid material for SSF has been suggested by Pandey et al. (1995) due to its high nutritional values [19]. In the present investigation, the potential application of SSF process in directly producing MCFAs from coconut fat has been studied. This process, generally named as direct modification of substrate through fermentation (DIMOSFER), was applied for the first time in oil modification. Therefore, the idea of this work would be to use a GRAS microorganism (*G. candidum*) to produce GRAS antimicrobial agents (MCFAs) particularly lauric acid through a green, clean, and cost-effective method (DIMOSFER).

## 2. Materials and Methods

### 2.1. Microorganism

Local* G. candidum* strain was purchased from Malaysia Agriculture Research and Development Institute (MARDI, Serdang, Selangor, Malaysia). The fungus was maintained on potato dextrose agar (PDA) slants at 4°C and periodically subcultured.

### 2.2. Inoculum Preparation

Inoculum suspension was prepared from the fresh, mature culture (7 days old at 30°C) of local *G. candidum* on potato dextrose agar slant. The spores were harvested with sterile distilled water containing 0.01% tween 80, transferred to a sterile tube, and the resulting suspension was homogenized for 15 s with a gyratory vortex mixer at 2000 rpm. Appropriate concentration (inoculum size = 10^5^ spores/mL), counting in a cell-counting haemocytometer, was inoculated into potato dextrose broth (PDB) (modified from [20]). 

### 2.3. Solid-State Fermentation and Optimization of MCFAs Production

Solid-state fermentation was carried out in 250 mL conical flask containing 10 g of coconut flakes [19]. Independent variables and ranges were selected based on the preliminary studies, where the level of moisture content, which was adjusted by distilled water, varied from 10 to 50%, and the level of external coconut oil content varied from 0 to 50% (v/w). The flasks were sterilized by autoclaving at 121°C for 20 min. After cooling down, the flasks were inoculated with 2 mL of 3-day-old PDB culture of local *G. candidum* strain. The content of each flask was mixed thoroughly with sterile spatula for uniform distribution of fungal spores in the medium. Flasks were incubated for a period of 3 to 30 days at 30°C. Samples were withdrawn for analysis according to the experimental design (five-levels three factor central composite rotary design, CCRD) at different periods of time (Table 1). Samples were then kept in the freezer for oil extraction and chemical analysis. Response surface methodology (RSM) was employed to build the best model and optimize the fermentation system using Design Expert version 6.06 (Stat Ease Inc. USA) (Table 1). One-way ANOVA was employed to study the main effects and intractions between parameters selected on coconut oil bioconversion.

### 2.4. Lipolytic Activity: A Time Course Study

Fermentation was carried out in 250 mL conical flasks each containing 10 g of coconut flakes, where the effective parameters were adjusted at the point of maximum MCFAs production. The flasks were incubated at 30°C and harvested every 24 h (for the period of 40 days). In order to evaluate the extracellular lipolytic activity [21], the content of each flask was soaked with 100 mL of aqueous solution of phosphate buffer 100 mM, pH 7 and shaken on a rotary shaker (200 rpm) for 1 h at 30°C. Finally, the suspension was squeezed through a double-layer muslin cloth and solution was centrifuged at 4000 ×g for 20 min at 4°C, and the supernatant was filtered through a membrane filter (pore size of 0.22 *μ*m). The clear filtrate obtained was assayed for extracellular lipolytic activity [22]. Furthermore, intracellular lipolytic activity was assayed after breakage of the cells using different common methods and followed by filtration. The clear filtrate obtained was assayed for intracellular and cell debris on the filter for cell-bound lipolytic activities. Cell breakage efficiency was assessed using microscopic tests. One unit of lipolytic activity was defined as 1.0 *μ*mol of free fatty acid liberated min^−1^ and reported as Uml^−1^. All reported data were the average of triplicate experiments.

### 2.5. Product Characteristics Analysis

#### 2.5.1. Coconut Oil Extraction from Solid Culture

Fermented coconut samples were kept in freezer after ending the incubation period based on the CCRD. Subsequently, 2 g of samples from each flask was placed in the round bottle of the soxhlet extractor. Petroleum ether (200 mL) was added to each sample, and oil extraction was performed for 10 h under moderate temperature (40–50°C). The solvent from oil/solvent mixtures was evaporated to get the extracted coconut oil after fermentation, and the product is called modified coconut oil (MCO). All treatment combinations were conducted in triplicates.

#### 2.5.2. Acylglycerol Composition Analysis

The acylglycerol composition of the extracted oil sample was determined by using reverse-phase high-performance liquid chromatography (RP-HPLC) (Alliance model Waters e2695 Separation Modules, UK) equipped with ELSD (Alliance model Waters 2424 ELS Detector, UK). Samples were dissolved in acetone (5% v/v) and after filtration through a 0.45 *μ*m PTFE membrane filter were injected onto Merck KGaA (Darmstadt, Germany) LiChrospher 100 RP-18e 5 *μ*m (250 mm × 4 mm) column under gradient condition [23]. The mobile phase used was a gradient of acetone and acetonitrile mixture (from 90% acetonitrile-10% acetone to 85% acetonitrile-15% acetone within first 15 min, then to 20% acetonitrile-80% acetone within next 20 min; and to 90% acetonitrile-10% acetone for last 10 min), where the flow rate was adjusted at 1 mL/min. The column temperature was maintained at 35°C. The drift tube and nebulizer of detector were set at 55°C and 36°C, respectively. The nitrogen gas pressure was 35 psi and the total time for a HPLC run was 45 min. The retention time was 3–7 min for FFA and MG peaks, 8–18 min for DG peaks, and 23–37 min for TG peaks. Each fraction was quantified based on the area normalization approach. TG peaks were identified based on the retention time of TG standards. Each sample was analyzed three times, and the data were reported as mean ± SD of percentage areas.

#### 2.5.3. Antibacterial Activity Studies

Antibacterial activity of the modified coconut oils was evaluated using both Gram-negative and Gram-positive bacteria. Selected Gram-negative bacterium *Escherichia coli *(ATCC 10536)  and Gram-positive bacterium *Staphylococcus aureus* (ATCC 25923) were cultivated aerobically at 37°C for 12 h in trypton soy broth (TSB) medium. Bacterial inoculums were prepared at the midlogarithmic phase of their growth containing approximately 10^8^ colony-forming units per mL (cfu/mL). It was achieved by diluting the overnight cultures of bacteria with the fresh TSB medium until constant absorbance at 630 nm was gained (OD_630_ = 0.5) [24].

Antibacterial activity of the modified oils was evaluated following the method described by Patgaonkar et al. (2011) [24] with some modifications by Ghanbari et al. (2012) [25]. The sample was prepared by mixing the bacterial inoculum (10 *μ*L), TSB medium (120 *μ*L), and modified oil (120 *μ*L) in each well of the 96-well microplate in triplicates. Control samples contained media and bacterial culture, with and without oil (nonmodified). After incubation of samples at temperature of 37°C for 12 h, their absorbance was measured at 650 nm using microplate reader (Power wave, Biotek). The percentage of inhibition was calculated as [(OD_control_ − OD_sample_)/OD_control_) × 100].

## 3. Results and Discussion

### 3.1. Lipolytic Activity: A Time Course Study

In order to study the direct hydrolysis process of coconut oil through SSF (DIMOSFER process), the lipolytic activity of the culture was evaluated in a time course study. Results demonstrated no extracellular lipolytic activity. Therefore, the high rate of hydrolysed coconut oil could be associated with intracellular or cell-bound lipolytic activities [26]. However, no lipolytic activity was detected in the cell filtrate after cell disruption using homogenization, sonication, and normal solvent extraction methods, and only cell-bound associated lipolytic activity was responsible for *in situ* coconut oil modification. Likewise, a membrane bound lipase in *G. candidum* GC-4 [27] and a mycelial lipase in a *G. candidum* strain [28] have been reported.

### 3.2. Modeling and Optimization of DIMOSFER Process for MCFAs Production

Hydrolysis of coconut oil by local *G. candidum* lipolytic activity in SSF was studied, and the yield of corresponding MCFAs production was optimized using response surface methodology (RSM). The selected effective variables were moisture content (%), coconut oil (%), and incubation period of time (day). Shredded coconut meat as an oily source was used as solid support in the solid culture, which originally contained 33.5% of oil (internal coconut oil) and 50% of moisture (internal moisture content). Similarly, the same levels of oil and moisture contents in the coconut were reported by Pandey et al. (1995) [19].

Among effective parameters chosen, moisture content was very essential for this *G. candidum* growth and coconut oil hydrolysis in DIMOSFER process. Based on preliminary study, the original moisture content (internal moisture) of the coconut flakes was not sufficient for direct hydrolysis reaction of the oil. Therefore, additional water (10–50% v/w) was added into the coconut-based medium (external moisture). The solid culture oil content was the second essential parameter in this study. Coconut flakes considered as oily substrate originally contained 33.5% oil in their parenchyma (internal oil). Additional oil (external oil) would be necessary for more efficiently hydrolysis by the fungal lipolytic activity in the solid culture and eventually to produce more MCFAs. The external coconut oil level was 0 to 50% (v/w), and incubation period of time was 3 to 30 days based on the preliminary study. The rate of coconut oil hydrolysis in DIMOSFER process was reported as response in this modeling and optimization study (Table 1). The extracted modified coconut oils after fermentation processes (based on the conditions of CCRD), which contained less triglycerides and more FFAs, have been shown in Table 1.

A reduced cubic model (1) was found to be the best-fitted model to explain the functionality of the system. Coefficient of determination (*R* ^2^ = 0.8728) and significant *F*-test analysis (*F*_model_ = 4.99) and probability value (*P*_model_ > *F* = 0.0154) indicated that the model was highly reliable. The model also showed insignificant lack of fit as shown by probability value (*P*_model_ > *F* = 0.6705) at 5% significance threshold for MCFAs-rich coconut oil production (Table 2).

Final equation obtained from the reduced cubic model to get the maximum coconut oil hydrolysis in DIMOSFER process (after fermentation) was as follows (1):

(1) [Coconut oil hydrolysis  (%)=−7144.59  +207.66M−4.64O+332.75t  −1.83M2+0.047O2−3.15t2−7.43M·t+0.12O·t+4.41E−3M3+0.064t3+0.047M2·t],

where *M*, *O*, and *t* were moisture content (%), oil content (%), and incubation time (day), respectively.

#### 3.2.1. Main Effects and Interactions between Parameters

**Figure 1:**
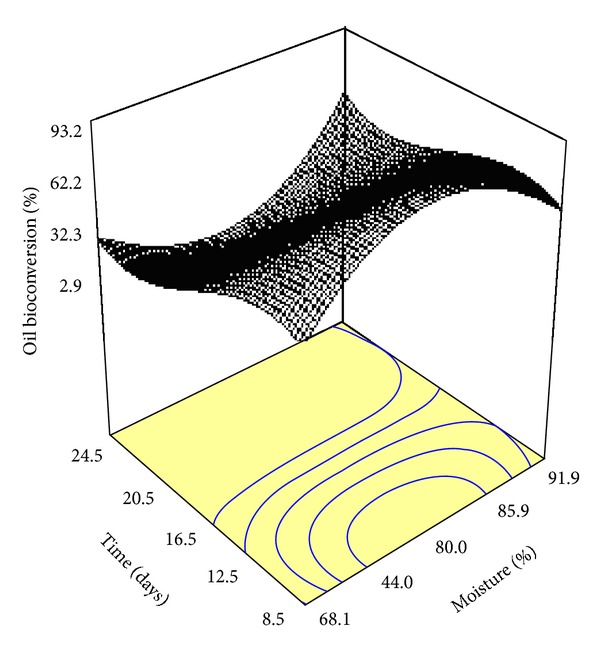
Three-dimensional graphs for the solid-state coconut oil hydrolysis by local *G. candidum*.

Figure 1 shows the interaction between moisture content (*M*) and incubation time (*t*) on coconut oil hydrolysis by local lipolytic *G. candidum* through SSF. The moisture parameter revealed significant changes in yields of oil hydrolysis. By increasing the moisture content, the rate of coconut oil hydrolysis increased significantly until *M* reached to the middle of the applied range. Increasing *M* after the middle value decreased the yield of response drastically. On the other hand, the yield of coconut oil hydrolysis was maximized at the beginning of incubation period (time) while, after that, the response decreased slightly. Based on the model achieved and its related ANOVA, *M* was more effective than *t* on the oil bioconversion.

Other studies [29, 30] also demonstrated that the optimum moisture level in SSF has a great impact on the physical properties of the solid substrate as well as the enzyme production. It has been stated that lower moisture than optimum decreases the solubility of the solid substrate, lowers the degree of swelling, and produces a higher water tension. Likewise, higher moisture levels than optimum value cause decreased porosity, lower oxygen transfer, and alteration in solid-state particle structure [29]. In our previous study, RSM revealed good understanding in complicated biological systems [31]. A reduced cubic-fitted model (Table 2) revealed its potential to determine the best operative conditions for *G. candidum* local strain lipolytic activity towards coconut oil hydrolysis and MCFA production. Finally, six different conditions of MCOs (MCO_1_–MCO_6_) were compared together in the case of antibacterial activity (Table 3).

#### 3.2.2. Optimum Condition

**Figure 2:**
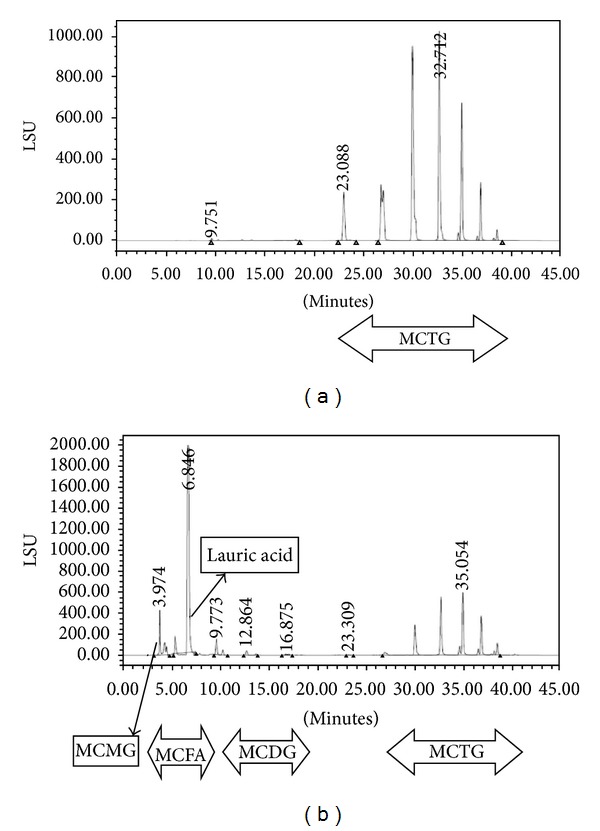
Coconut oil (a) and optimum modified coconut oil (MCO_6_), (b) glycerides profiles analyzed by ELSD-RP-HPLC.

In order to obtain the maximum lipolysis and MCFAs production in DIMOSFER, process conditions were optimized. The optimum lipolytic function of local* G. candidum* on coconut oil was found to be 29% moisture content and 10.14% oil content, after 9 days of incubation. Maximum coconut oil hydrolysis was 76% under the optimum condition, which consisted of 53% total MCFAs. As shown in Figure 2, the level of medium chain triglycerides (MCTG) content dropped after fermentation, where medium chain monoglycerides (MCMGs), medium chain diglycerides (MCDGs), and medium chain fatty acids (MCFAs) were produced. However, the level of generated lauric acid was obviously the highest compared to other compounds (Figure 2). Hence, the function of this local nonextracellular* G. candidum* lipase in SSF on coconut oil hydrolysis was effective.

The optimum values obtained for this fungus in the solid culture indicated that the local *G. candidum* was able to produce MCFAs under economic conditions. The optimum oil content of the solid culture was found to be 10.14%, which was less than the center level (25%). Time was kept at 9 days after incubation, which was also lower than the center point (16.5 days) to obtain maximum level of coconut oil hydrolysis. Our finding also showed that the optimum level of moisture content required was 29% which was close but still lower than the center point (30%). Thus, from an industrial point of view, to get the optimum lipolytic reaction at the lowest possible level of time, the local *G. candidum* strain could be a good choice as all parameters were optimized at low amounts. Based on our observation, the low water activity required could improve the function of local *G. candidum*, where this characteristic is very crucial in large-scale production to prevent a sticky culture [29].

### 3.3. Feasibility of Direct Modification of Coconut Oil Process

According to the results obtained in this work, DIMOSFER process could be applied for modification of oil substrate in SSF. As shown in Figure 2, MCTGs content of coconut oil was partially hydrolysed into MCMGs, MCDGs, and particularly MCFAs after fermentation process, where eventually coconut oil turned into the MCAFs-rich coconut oil. To the best of our knowledge, the feasibility of this method in any oil modification and fatty acid production has never been reported. Fernandes et al. (2004) [32] and Martínez-Ruiz et al. (2008) [33] used dried SSF preparations as economical biocatalysts for synthetic reactions in organic solvents. They demonstrated the feasibility of using dried fermented solids, containing lipases without expensive extraction, purification, and immobilization processing. Moreover, Parfene et al. (2013) [34] produced MCFAs using yeast lipase through an agar-based solid culture on plate. The use of DIMOSFER process in a natural plant-based solid culture for MCFAs production using lipolytic *G. candidum* strain was examined for the first time in this study. 

### 3.4. Antibacterial Effects of Modified Coconut Oils (MCOs)

Based on the suggested conditions by the reduced cubic model, MCO_1_ to MCO_6_ in Table 3 were compared to the normal coconut oil. The results demonstrated that all MCOs (MCO_1_–MCO_6_) extracted from the local* G. candidum* solid cultures revealed significant improved levels of antibacterial activities (ABAs) compared to the control (Table 3). MCOs, which were produced from hydrolysis of coconut oil during DIMOSFER process, contained different proportions of MCTGs, MCDGs, MCMGs, and MCFAs (Figure 2(b)) compared to the normal coconut oil, which contained MCTGs (Figure 2(a)). Detailed analysis of the lipid classes by RP-ELSD-HPLC (Figure 2) showed that the amount of MCFAs particularly C_12_ (lauric acid) played important role in inactivation of the growth of both Gram-positive (*Staphylococcus aureus*) and Gram-negative (*E. coli*) bacteria selected (Table 3). Similarly, results obtained by Carroll (1980) [35] indicated that high amount of fatty acids content play important role in broadening the antimicrobial spectrum of modified oils. In addition, it has been demonstrated that the MCFAs and their corresponding monoglycerides and diglycerides have antimicrobial effects against *S. aureus* [7, 36] and *E. coli* [37–40]. Hayashi (1995) [38] indicated that the combination of MCTG, MCDG, MCMG, and MCFA revealed broad range of antimicrobial properties against human pathogens and enveloped viruses. Moreover, these compounds are known to have antimicrobial effects against food-borne pathogens like *L. monocytogenes* [41] and *C. botulinum *[42].

As shown in Table 3, the level of MCFAs, mostly composed of free lauric acid, was the most important factor for antibacterial activity. The highest antimicrobial activities (90% against *S. aureus* and 80% against *E. coli*) obtained under maximum level of MCFAs (53%) produced particularly lauric acid. The effectiveness of free lauric acid in antibacterial activity against Gram-positive and Gram-negative bacteria was also demonstrated by Khoramnia et al. (2013) [43]. It has been well established that lauric acid represents the strongest antimicrobial activity among all fatty acids [44].

Sado-Kamdem et al. (2009) [3] indicated that the antimicrobial mechanism of MCFAs is to increase cell membrane fluidity when added to foods. As stated by Sado Kamdem et al. (2008) [2], FFAs' affect the division intervals of single cells which bring about an inhibitory effect compared to the control medium's longer division intervals. The antimicrobial action of lauric acid is due to the penetration of acid in the lipid membrane of the bacterial cell. The corresponding cellular acidic pH leads cell death by suppressing cytoplasmic enzymes and nutritional transport systems as well as uncoupling ATP driven pumps [45, 46]. Similar mechanism has been proposed for MCFA, MCDG, and MCMG [40], whereby these functional lipids kill bacteria by disrupting the permeability barrier of cell membrane. For instance, it was reported that lauric acid-rich feed prevented the death of infected mice, Guinea pigs, and cows by inhibiting the growth of *Mycobacterium* by interfering with the enzymatic systems [35].

Nakatsuji et al. (2009) [47] found that lauric acid has the potential of becoming an innate, safe, and effective therapeutic medication for all *P. acnes-*associated diseases. The safety of lauric acid and its esters when consumed in large doses and over extended periods of time was indicated to be safe [4]. Therefore, they could be considered as natural GRAS antimicrobial components. Since coconut oil has been widely used in cosmetic products and also approved for clinical applications, this lauric acid enriched oil developed from this work has the potential to be used in therapeutic applications. Similarly Hristov et al. (2009) [9] indicated that combination of lauric acid and coconut oil revealed even higher antimicrobial activity, better milk fatty acid alteration, and lower methane production *in vivo* compared to the individual application of these elements.

Moreover, as stated by Kitahara et al. (2006) [48], MCFAs particularly lauric acid are suitable for external application for infection control and medical treatment in hospitals. Soni et al. (2010) [8] reported that application of lauric acid in cheese enhanced bactericidal activity without affecting sensory quality. In addition, Soni et al. (2012) [49] illustrated that the mixture of MCFAs enhanced the antimicrobial activity and methane formation suppression in ruminants effectively. All these studies are testament to the potential application of the produced MCO.

## 4. Conclusions

In the present study, the use of DIMOSFER in production of value-added coconut oil by a Malaysian strain of lipolytic *G. candidum *and the chemical characterization of the modified oils and their antimicrobial activities were investigated. The variation in glycerides composition of MCOs and the extracted coconut oils after fermentation process was evaluated by ELSD-RP-HPLC analysis. The yield of coconut oil hydrolysis and MCFAs production through DIMOSFER process were optimized by a reduced cubic model at 76% and 53%, respectively. The fungal lipolytic activity on coconut fat hydrolysis was maximized at 29% of moisture content and 10.14% of oil content after 9 days of incubation in SSF. Antimicrobial activities of MCOs were evaluated against some food-borne bacteria, and an increase in inhibitory activity with increasing concentration of MCFAs particularly lauric acid was noted. Therefore, DIMOSFER process accompanied with the use of a novel local lipolytic *G. candidum *as a GRAS microorganism can be considered as a “green” process. This process was found to be advantageous in MCFAs production as GRAS antimicrobial agents. The produced MCOs, rich in free MCFAs content, could be further applied for food, cosmetic, and pharmaceutical purposes. Natural enrichment of lauric acid in an edible culture offers a new approach to increase lauric acid intake in human populations with the potential to improve long-term human health.

## Figures and Tables

**Table 1:** CCRD for coconut oil hydrolysis and the level of derivatives in the form of MCFA, MCDG, and MCMG produced by local *G. candidum* lipase in SSF.

Run no.	Moisture (additional) (%)	Oil (%)	Time (day)	Coconut oil hydrolysis (%)	MCFA (%)	MCDG (%)	MCMG (%)
1	18	10	16.0	40.00	6.70	26.00	7.32
2	42	10	16.0	62.00	480	7.30	6.74
3	18	40	8.5	7.67	3.67	3.85	0.16
4	42	40	8.5	2.43	0.17	2.36	0.00
5	18	10	24.5	7.40	1.24	6.15	0.00
6	42	10	30.0	46.00	22.40	20.50	2.64
7	18	40	24.5	13.12	4.64	8.48	0.00
8	42	40	24.5	27.30	18.30	9.00	0.00
9	10	25	16.5	30.00	7.77	21.60	0.70
10	50	25	16.5	55.80	47.00	7.80	1.00
11	30	0	16.5	54.13	40.00	6.78	7.43
12	30	50	16.5	39.70	14.18	14.80	0.63
13	30	25	3.0	1.00	0.00	1.00	0.00
14	30	25	30.0	61.00	46.40	14.30	0.50
15	30	25	16.5	28.00	7.50	20.30	0.22
16	30	25	25.0	11.42	2.10	9.32	0.00
17	30	25	16.5	28.00	17.80	9.70	0.50
18	30	25	16.5	14.73	7.73	7.00	0.00
19	30	25	16.5	20.00	7.00	13.00	0.00
20	30	25	16.5	15.00	5.00	10.00	0.00

**Table 2:** ANOVA analysis of reduced cubic model.

Source	Sum of squares	DF	Mean square	*F* value	Prob > *F*	
Model	6489.22	11	589.93	4.99	0.0154	Significant
*A*	7.03	1	7.03	0.059		
*B*	265.65	1	265.65	2.25		
*C*	601.29	1	601.29	5.09		
*A* ^2^	0.061	1	0.061	5.14*E* − 04		
*B* ^2^	1262.8	1	1262.8	10.68		
*C* ^2^	45.34	1	45.34	0.38		
*AC*	271.67	1	271.67	2.3		
*BC*	1012.97	1	1012.97	8.57		
*A* ^3^	215.65	1	215.65	1.82		
*C* ^3^	804.19	1	804.19	6.8		
*A* ^2^ *C*	568.14	1	568.14	4.81		
Residual	945.57	8	118.2			
Lack of fit	234.22	3	78.07	0.55	0.6705	not significant
Pure error	711.34	5	142.27			
Cor total	7434.79	19				

*A*: Moisture content (*M*); *B*: oil content (*O*); *C*: time (*t*).

*R*-squared = 0.8728; Adj *R*-squared = 0.8044.

**Table 3:** Composition of the selected modified coconut oils (MCO_1_–MCO_6_) produced by local *G. candidum* through DIMOSFER process along with their antibacterial activities.

Sample	SSF condition			Product composition (%)				ABA (%)	
	Moisture content (%)	Oil content (%)	Incubation time (day)	MCTG	MCDG	MCMG	MCFA	*E. coli *	*S. aureus *
MCO_1_	32	50	30	80	15	1	14	15	20
MCO_2_	42	10	10	54	20.52	2.64	22.4	71	75
MCO_3_	30	0	16	54.5	9.8	4.65	31.14	60	63
MCO_4_	30	25	10	38	20.32	0.5	40.5	75	80
MCO_5_	50	25	16	44.5	7.8	1	47	78	85
MCO_6_ (opt)	30	10	9	24	16.55	6.45	53	80	90
Coconut oil				99	1	0	0	3.1	4.8
Without oil				—	—	—	—	0*	0*

MCO_1_–MCO_6_: modified coconut oils obtained from DIMOSFER by local *G. candidum* strain.

Opt: optimum condition obtained from optimization of coconut oil hydrolysis through DIMOSFER process.

Composition: MCTG: medium chain triglyceride; MCDG: medium chain diglyceride; MCMG: medium chain monoglyceride; and MCFA: medium chain fatty acid.

ABA: antibacterial activity.

Pathogenic bacteria: *Escherichia coli *(ATCC 10536) and *Staphylococcus aureus* (ATCC 25923).

^∗^ *Escherichia coli *(1.44 × 10^8^ CFU/mL) and *Staphylococcus aureus* (1.30 × 10^8^ CFU/mL).
